# A patient with heatstroke associated with consciousness disturbance secondary to hyponatremia: a case report

**DOI:** 10.1186/1752-1947-7-62

**Published:** 2013-03-08

**Authors:** Masaki Miyasaka, Tamae Hamaki, Masahiro Kami, Yuuichi Hamabe

**Affiliations:** 1 Tokyo Metropolitan Bokutoh General Hospital, 4-23-15, Kohtohbashi, Sumida-ku, Tokyo, Japan; 2 Division of Social Communication System for Advanced Clinical Research, Institute of Medical Science, The University of Tokyo, 1-4-6, Shirokanedai, Minato-ku, Tokyo, Japan

## Abstract

**Introduction:**

Little information is available on the pathogenesis of heatstroke without strenuous exercise in younger patients. Here, we report the case of a 31-year-old man who developed heatstroke secondary to hyponatremia. His condition was initially misdiagnosed as classic heatstroke. We hope the detailed description of our patient’s case will provide valuable information for medical professionals faced with similar cases in the future.

**Case presentation:**

A 31-year-old Japanese man who was regularly taking anti-hypertensive agents, including a thiazide, was admitted to our hospital owing to consciousness disturbance and a high-grade fever. A thorough examination revealed mild renal dysfunction, rhabdomyolysis, a hyper-coagulable state, and severe hyponatremia (114mEq/L). Because he worked in a hot environment, an initial diagnosis of heatstroke was established. His general condition improved rapidly with supportive measures, and he was discharged on the fourth day after admission. Interestingly, he did not recognize feeling hot while working in a hot environment. These findings suggest that the consciousness disturbance, which was probably attributable to acute hyponatremia caused by the thiazide, preceded the onset of the heatstroke.

**Conclusions:**

Clinicians should consider the presence of underlying diseases, especially consciousness disturbance, when younger individuals develop heatstroke in the absence of strenuous exercise.

## Introduction

Heatstroke is a life-threatening illness characterized by an elevated core temperature of >40°C and dysfunction of the central nervous system, resulting in delirium, convulsions and coma [1].

There are two types of heatstroke: classic and exertional. The classic type affects individuals, most often older patients, with underlying chronic medical conditions [2]. The exertional type generally occurs in younger, otherwise healthy individuals in heavy exercise scenarios under high ambient temperature and humidity. Typical patients are athletes and military recruits in basic training [3].

There is little information on the pathogenesis of heatstroke without high intensity exercise in younger individuals. Here, we report the case of a 31-year-old man whose condition was diagnosed as heatstroke attributed to doing light work in a hotel room. We hope the detailed description of our patient’s case will provide valuable information for medical professionals facing similar cases in the future.

## Case presentation

A 31-year-old Japanese man who worked as a janitor was admitted to Tokyo Metropolitan Bokutoh General Hospital for coma and convulsion in September 2010. During this summer, an exceptional heat wave was experienced in Japan, during which 1718 individuals died from heatstroke [4].

Until admission, he had conducted a non-strenuous work in a hot environment for one or two hours. A work colleague found him staggering in the corridor and moved him to a cool room, where he developed convulsions lasting for 15 seconds.

A diagnosis of essential hypertension had been established at the age of 26, when a combination treatment of 50mg losartan and 12.5mg hydrochlorothiazide, and doxazosin mesilate at 1mg were initiated. Since control of his blood pressure became poor, eplerenone at 50mg was initiated five days before admission.

Prior to his admission, no abnormal findings other than hypertension and obesity, with a body mass index of 27kg/m^2^, had been detected on routine physical examinations and laboratory testing. Repeated blood tests failed to show any abnormal findings in serum electrolyte levels.

His initial evaluation showed consciousness disturbance and a Glasgow Coma Scale level of 10 (E4V2M4). The temperature in his urinary bladder was 40.6°C. His blood pressure was 116/52torr, and his heart rate was 157 beats/minute. His respiratory rate was 34 breaths/minute.

The initial laboratory test findings are shown in Table 1. Abnormal findings comprised serum levels of creatinine 1.4mg/dL, sodium 114mEq/L and creatine kinase 756IU/L. He met the criteria for systemic inflammatory response syndrome [5].

**Table 1:** Laboratory tests and results

**Laboratory finding**	**First day**	**Second day**	**Third day**	**Fourth day**	**20th day**
Blood urea nitrogen (mg/dL)	17	22		7	17
Creatinine (mg/dL)	1.4	0.7		0.5	1.4
Creatine kinase (U/L)	756	2866	2257	1126	756
Uric acid (.mg/dL)	7.8	5.1		2.4	7.8
Bicarbonate (mEq/L)	11.9	-	-	-	
Free T4 (ng/dL)	1.16	-	-	-	1.19
TSH (μU/mL)	0.4	-			0.59
Cortisol level (μg/dL)	-				6.6
Serum sodium (mEq/L)	114	124	126	127	
Urine sodium (mEq/L)	107	84			
Hemoglobin A1c (percent)	5.4				
Blood glucose (mg/dL)	218				
Platelet count (10^4^ cells/mm^3^)	18.6				
PT/INR	0.97				
APTT (seconds)	21.8				
Fibrinogen (mg/dL)	437				
D-dimer level (μg/mL)	7.4				

The first, second and third days were during admission. Our patient was discharged on the fourth day.

APTT, activated partial thromboplastin time; PT/INR, prothrombin time/international normalized ratio; TSH, thyroid stimulating hormone.

Except for severe hyponatremia of 114mEq/L, there were no abnormal findings leading to the development of consciousness disturbance. Serum levels of thyroid hormones and cortisols were normal. The results of blood culture tests were negative. No signs suggesting the presence of sepsis were documented. A computed tomography scan of our patient’s head failed to show any abnormal findings.

Based on these findings, the initial diagnosis was heatstroke complicated by dehydration, rhabdomyolysis, hyponatremia, renal failure and a hyper-coagulable state.

The initial treatment comprised cooling, intravenous fluid administration, tracheal intubation, and mechanical ventilation. The anti-hypertensive drugs and diuretics were withdrawn. We administered 5700mL of normal saline intravenously during the first nine hours. His urine output was 2000mL during the same periods. He was additionally given 1920mL of normal saline during the next 24 hours, when 3000mL of urine was excreted.

Our patient’s levels of consciousness rapidly recovered to 12 (E4V3M5) with his core body temperature returning from 40.6°C to 38.2°C within three hours of the initiation of cooling and intravenous fluid.

On the next day, he became fully conscious without development of central pontine myelinolyis. His serum sodium concentration improved from 114mEq/L to 124mEq/L within 18 hours. His core body temperature returned to normal (36.9°C). His general condition improved rapidly, and he was discharged on the fourth day.

Eplerenone was resumed and the hyponatremia did not recur. Because hyponatremia is a common complication of thiazides, we withheld the losartan/hydrochlorothiazide. Challenge tests were omitted because our patient did not agree to undertake them.

After he recovered, we took another careful review of our patient’s medical history. Surprisingly, he did not recognize feeling hot while working in a hot environment. Considering his clinical course, a diagnosis of heatstroke preceded by consciousness loss due to severe hyponatremia was made.

## Discussion

Our patient’s case indicates that some underlying disease might exist in younger patients who develop heatstroke during light work. The heatstroke in our patient’s case was probably preceded by hyponatremia with disturbed consciousness. Considering that our patient could remember what he was working on at the time, retrograde amnesia seemed unlikely. Clinicians should suspect the presence of underlying disease when younger patients develop heatstroke without strenuous exercise.

It is important to identify the cause of consciousness disturbance. Severe hyponatremia with a serum sodium level of 114mEq/L was documented upon our patient’s admission. Because coma is a common complication of severe hyponatremia, it is reasonable to assume that the disturbance of consciousness was associated with hyponatremia.

Although hyponatremia is often reported in patients with heatstroke [6], most physicians believe that overdrinking to compensate for dehydration leads to the dilution of serum sodium. However, this was unlikely in our patient because he was not able to drink much water during his work. Based on a careful history-taking from our patient and his family after discharge, we estimated that the amount was less than 500mL.

Considering his clinical course, asymptomatic hyponatremia might have existed at the beginning of his work. We can consider some possibilities. Five days after eplerenone had been initiated, severe hyponatremia was documented. His clinical course in chronological order suggests a possible association between eplerenone and hyponatremia, although few reports have been published on hyponatremia due to eplerenone.

The hydrochlorothiazide he was taking played an important role. Thiazides are a common cause of severe hyponatremia [7]. Water retention from impaired water excretion combined with cation depletion, which thiazides induce, may result in severe hyponatremia [8]. The additional eplerenone might have influenced the pharmacokinetics of thiazide leading to the development of severe hyponatremia.

However, most patients with thiazide-induced hyponatremia are older [7]. Further studies are warranted to clarify why hyponatremia developed in our patient. Some genetic factors might also be involved in the pathogenesis.

## Conclusions

Clinicians should consider the presence of underlying disease, especially in cases of consciousness disturbance, when younger patients develop heatstroke in the absence of strenuous exercise. Furthermore, if they show signs of hyponatremia, clinicians should suspect its possible association with consciousness disturbance.

## Consent

Written informed consent was obtained from the patient for publication of this report and any accompanying images. A copy of the written consent is available for review by the Editor-in-Chief of this journal.

## Competing interests

The authors declare that they have no competing interests.

## Authors’ contributions

MM wrote the manuscript, followed our patient, collected the data and was responsible for literature research. KM, TH, YH were involved in critically revising the manuscript for important intellectual content. All authors read and approved the final manuscript.
